# American Health Primers

**Published:** 1879-08

**Authors:** Joseph G. Richardson


### AMERICAN HEALTH PRIMERS.

### LONG LIFE, AND HOW TO REACH IT.

*By Joseph G. Richardson, M. D.*

This is one of a series of popular treatises upon life and health, being published by Lindsay & Blakiston, of Philadelphia.

The following subjects are discussed, viz.:

Causes of Disease, and How to Avoid Them.

Heat and Cold as Causes of Disease.

Contagion, and How to Escape It.

Clothing, and How to Wear It.

Pure Air, and How to Breathe It.

Pure Water, and How to Obtain It.

Baths, and How to Take Them.

The House, and How to Build It.

Food, and How to Digest It.

Impurities in Food and Drink, and How to Detect Them.

Exercise, and How to Take It.

Sleep, and How to Secure It.

Mental Power, and How to Retain It.

Parasitic Enemies, and How to Escape Them.

Old Age, and How to Meet It.

These various subjects are briefly but clearly discussed, and very valuable information given, and suggestions made, which, if properly appreciated and followed, would be of inestimable benefit.

While not one in a thousand of the human family correctly apply the knowledge of life and health they already possess, yet, such presentations of the subjects as are given in this little work will certainly form a stimulus to higher activity.

This little work is most earnestly recommended to all who desire to do better and be better.

Other primers follow this, of which a note will be made.

